# Estimated number of deaths directly averted in people 60 years and older as a result of COVID-19 vaccination in the WHO European Region, December 2020 to November 2021

**DOI:** 10.2807/1560-7917.ES.2021.26.47.2101021

**Published:** 2021-11-25

**Authors:** Margaux MI Meslé, Jeremy Brown, Piers Mook, José Hagan, Roberta Pastore, Nick Bundle, Gianfranco Spiteri, Giovanni Ravasi, Nathalie Nicolay, Nick Andrews, Tetiana Dykhanovska, Joël Mossong, Małgorzata Sadkowska-Todys, Raina Nikiforova, Flavia Riccardo, Hinta Meijerink, Clara Mazagatos, Jan Kyncl, Jim McMenamin, Tanya Melillo, Stella Kaoustou, Daniel Lévy-Bruhl, Freek Haarhuis, Rivka Rich, Meaghan Kall, Dorit Nitzan, Catherine Smallwood, Richard G Pebody

**Affiliations:** 1 World Health Organization (WHO) Regional Office for Europe, Copenhagen, Denmark; 2 European Centre for Disease Prevention and Control (ECDC), Stockholm, Sweden; 3 UK Health Security Agency, London, United Kingdom; 4 Public Health Center of the MOH of Ukraine (UPHC), Kyiv, Ukraine; 5 Health Directorate, Luxembourg, Luxembourg; 6 National Institute of Public Health (NIH) – National Research Institute, Warsaw, Poland; 7 Centre for Disease Prevention and Control of Latvia, Riga, Latvia; 8 Instituto Superiore di Sanità, Rome, Italy; 9 Norwegian Institute of Public Health, Olso, Norway; 10 Institute of Health Carlos III, Madrid, Spain; 11 National Institute of Public Health, Prague, Czechia; 12 Public Health Scotland, Glasgow, United Kingdom; 13 Ministry of Public Health, Malta; 14 National Public Health Organization, Athens, Greece; 15 French Public Health Agency, Paris, France; 16 Sciensano, Brussels, Belgium; 17 Israel Ministry of Health, Jerusalem, Israel

**Keywords:** COVID-19, vaccination programs, older age groups, deaths averted, expected mortality, vaccination coverage

## Abstract

Since December 2019, over 1.5 million SARS-CoV-2-related fatalities have been recorded in the World Health Organization European Region - 90.2% in people ≥ 60 years. We calculated lives saved in this age group by COVID-19 vaccination in 33 countries from December 2020 to November 2021, using weekly reported deaths and vaccination coverage. We estimated that vaccination averted 469,186 deaths (51% of 911,302 expected deaths; sensitivity range: 129,851–733,744; 23–62%). Impact by country ranged 6–93%, largest when implementation was early.

Since the severe acute respiratory syndrome coronavirus 2 (SARS-CoV-2) was first detected in December 2019 [1], in excess of 1.5 million coronavirus disease (COVID-19) fatalities have been reported in the World Health Organization (WHO) European Region until week 45/2021, with 90.2% deaths in people 60 years and older [2]. In a subset of 33 countries in the Region covered by this study, 442,116 fatalities were reported between December 2020 and November 2021 in this age group. We observed a wide range in cumulative national mortality rate from 5.4 to 1,008.0 per 100,000 population 60 years and older. Here, we estimate the number of deaths averted in this age group since the start of COVID-19 vaccination in those countries of the WHO European Region with available data.

## Vaccination roll-out and uptake

**Figure 1:**
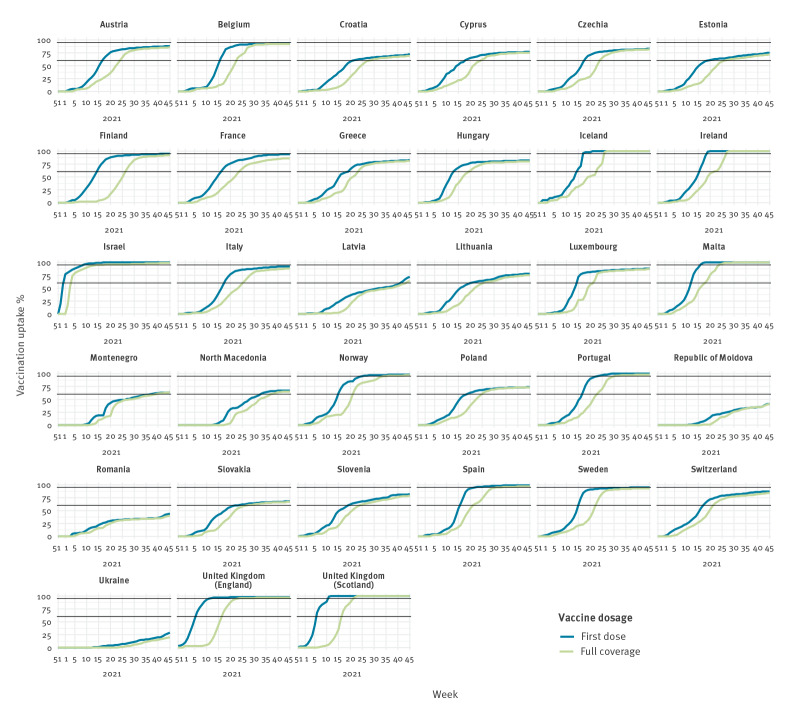
Cumulative vaccination coverage in the population aged 60 years and older, by country, 33 countries in the WHO European Region, weeks 51/2020–45/2021

**Figure 2:**
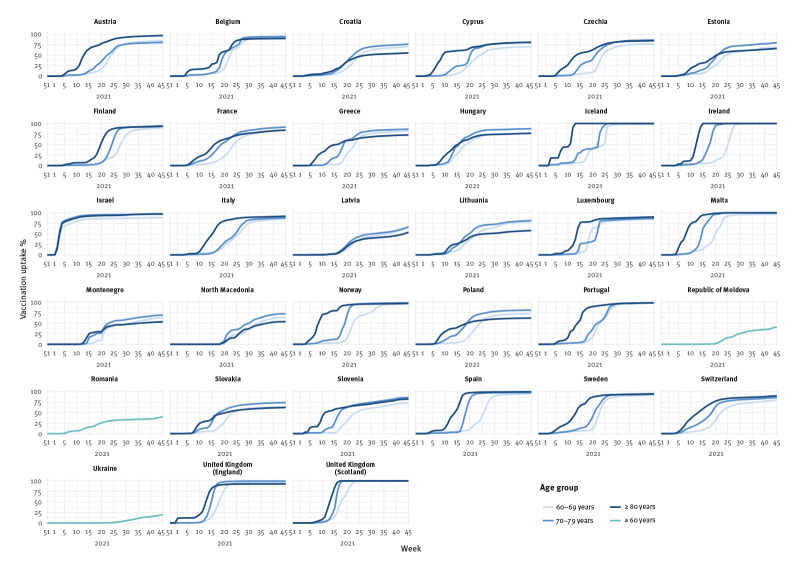
Complete vaccination coverage, by age group where available, in the population aged 60 years and older, by country, 33 countries in the WHO European Region, weeks 51/2020–45/2021

**Figure 3:**
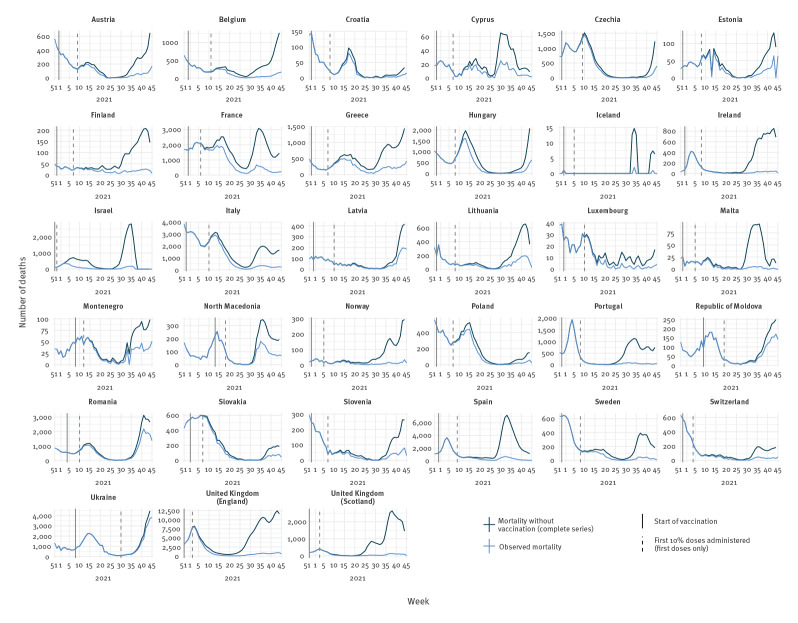
Observed and expected mortality, using the base vaccine effectiveness scenario^a^, together with timing of vaccination in population aged 60 years and older, by country, 33 countries in the WHO European Region, weeks 51/2020–45/2021

The emergence of SARS-CoV-2 was followed by the rapid development, licensure and roll-out of several COVID-19 vaccines from late 2020 onwards, initially targeting select groups including those at higher risk of severe disease, in particular older adults. Fifty-one countries, areas or territories in the Region had reported the weekly number of vaccination doses administered to The European Surveillance System (TESSy) until week 45/2021, of which 33 countries had reported age-specific mortality counts and number of vaccination doses administered. Vaccination uptake (VU) in these countries increased in priority groups such that by week 45, 80% (range: 20–100) of people 60 years and older had received a complete vaccination series and 84% (range: 29–100) had received at least one dose (Table 1, Figures 1, 2, 3). 

**Table 1:** Cumulative number of deaths observed and averted by COVID-19 vaccination, mortality rates and expected mortality rates per 100,000 population aged 60 years and older, using the base vaccine effectiveness scenario^a^, by country, WHO European Region, weeks 51/2020–45/2021

Country	Vaccines used	Vaccination uptake (%)		Number of deaths				Mortality rate per 100,000		
		Partial	Full	Observed	Averted after one dose	Averted after two doses	Averted total	Observed	Total expected	% expected deaths averted by vaccination
Iceland	AZ-COM-JANSS-MOD	100	100	4	0	52	52	5.4	76.0	93
United Kingdom (Scotland)	AZ-COM-MOD	100	100	4,585	454	27,202	27,656	333.3	2,343.8	86
Israel	AZ-COM-MOD	97	93	3,972	925	14,737	15,662	263.1	1,300.7	80
Norway	AZ-COM-JANSS-MOD	98	97	682	87	2,705	2,792	54.1	275.4	80
Ireland	AZ-COM-JANSS-MOD	100	100	3,156	116	8,958	9,074	325.5	1,261.2	74
Malta	AZ-COM-JANSS-MOD	100	100	305	26	834	860	245.4	937.3	74
Finland	AZ-COM-JANSS-MOD	95	92	1,007	282	2,327	2,609	62.7	225.1	72
Spain	AZ-COM-JANSS-MOD	99	97	34,032	2,102	87,413	89,515	277.1	1,006.1	72
United Kingdom (England)	UNK	98	97	74,354	14,918	142,686	157,604	557.1	1,738.0	68
Cyprus	AZ-COM-JANSS-MOD	77	75	530	51	603	654	221.8	495.5	55
Portugal	AZ-COM-JANSS-MOD	100	98	12,050	503	13,719	14,222	402.4	877.3	54
Austria	AZ-COM-JANSS-MOD	88	86	5,875	390	6,256	6,646	254.1	541.6	53
Greece	AZ-COM-JANSS-MOD	83	81	11,703	746	11,429	12,175	390.2	796.1	51
Belgium	AZ-COM-JANSS-MOD	93	92	7,708	775	7,046	7,821	259.8	523.3	50
France	AZ-COM-JANSS-MOD	94	86	47,681	5,732	32,983	38,715	272.2	493.1	45
Lithuania	AZ-COM-JANSS-MOD	78	75	4,155	176	3,244	3,420	555.6	1,012.9	45
Sweden	AZ-COM-MOD	95	93	6,612	487	4,283	4,770	252.3	434.4	42
Slovenia	AZ-COM-JANSS-MOD	82	79	2,798	122	1,626	1,748	485.2	788.3	38
Italy	AZ-COM-JANSS-MOD	92	88	60,898	3,900	31,588	35,488	337.5	534.2	37
Switzerland	COM-JANSS-MOD	87	84	4,703	167	2,476	2,643	214.9	335.6	36
Luxembourg	AZ-COM-JANSS-MOD	88	87	490	39	226	265	392.4	604.7	35
Estonia	AZ-COM-JANSS-MOD	75	72	1,290	69	604	673	362.4	551.4	34
Hungary	AZ-BECNBG-COM-JANSS-MOD-SPU	82	80	20,437	2,036	8,230	10,266	790.9	1,188.1	33
North Macedonia	AZ-BECNBG-COM-SIN-SPU	67	65	4,030	145	1,629	1,774	935.7	1,347.6	31
Montenegro	AZ-BECNBG-COM-SPU	64	64	1,399	26	555	581	1,008.0	1,426.6	29
Latvia	AZ-COM-JANSS-MOD	72	64	2,802	67	894	961	538.7	723.5	26
Czechia	AZ-COM-JANSS-MOD	83	82	20,292	980	4,607	5,587	724.5	924.0	22
Romania	AZ-COM-JANSS-MOD	44	40	30,250	299	7,223	7,522	606.3	757.0	20
Poland	AZ-COM-JANSS-MOD	74	73	8,241	382	1,610	1,992	83.9	104.2	19
Croatia	AZ-COM-JANSS-MOD	73	69	1,335	87	164	251	114.9	136.5	16
Slovakia	AZ-COM-JANSS-MOD-SPU	68	67	9,819	303	1,302	1,605	771.0	897.0	14
Moldova	AZ-BECNBG-COM-JANSS-SIN-SPU	41	41	3,584	13	514	527	470.3	539.4	13
Ukraine	AZ-COM-JANSS-MOD-SIICOV-SIN	29	20	51,337	561	2,495	3,056	496.5	526.0	6
**Total**		**84**	**80**	**442,116**	**36,966**	**432,220**	**469,186**	**365.2**	**752.8**	**51**

AZ: AstraZeneca Vaxzevria; BECNBG: Beijing CNBG BBIBP-CorV; COM: Pfizer BioNTech Comirnaty; JANSS: Janssen Ad26.COV 2-S; MOD: Moderna mRNA-1273; SPU: Gamaleya Gam-Covid-Vac; SIICOV: SII Covishield; SIN: Sinovac CoronaVac; UNK: unknown product; WHO: World Health Organization.

^a^ VE_1_ = 60% and VE_2_ = 95% and time lags of 4 and 3 weeks for first and second vaccination dose, respectively.

Countries are ordered according to the proportion of deaths averted.

WHO: World Health Organization.

Black horizontal lines represent 60% and 95% vaccination uptake respectively.

WHO: World Health Organization.

WHO: World Health Organization.

^a^ VE_1_ = 60% and VE_2_ = 95% and time lags of 4 and 3 weeks for first and second vaccination dose, respectively.

The number of observed deaths plotted here are the true numbers reported by the country and not the rolling average used in calculations.

The impact of such vaccination campaigns on preventing severe disease in terms of averted deaths can be quantified [3,4], although an assessment of lives saved using a standardised methodology across multiple countries in Europe is lacking. 

## Number of deaths averted as a result of vaccination among older adults

Data from 33 countries reporting both COVID-19 age-specific vaccination and age-aggregated COVID-19 mortality data for people 60 years and older were downloaded from TESSy on 18 November 2021 and restricted to the weeks between 51/2020 and 45/2021. Thirty countries reported more granular age groups (60–69, 70–79 and ≥ 80 years). Population estimates for each age group in year 2020 were drawn from the United Nations [5] for countries not in the European Union (EU) or European Economic Area (EEA) and from Eurostat [6] for EU/EEA countries, except for Israel (age denominators from Israel Central Bureau of Statistics [7]). All population data were downloaded in 2021. All analyses presented here were conducted in R software version 4.0.5 (R Foundation, Vienna, Austria) [8]. We assumed that all countries used the same definition for COVID-19 mortality and that reporting delays were comparable.

The weekly number of deaths averted per country was estimated using methods adapted from Machado et al. [9] (available on GitHub [10]), with the following definitions: **VE_1_** as vaccine effectiveness (VE) (at least one dose), **VE_2_** as vaccine effectiveness (complete series), **VU_1_** being vaccination uptake (at least one dose) and **VU_2_** as vaccination uptake (complete series). The rolling average number of deaths observed over three consecutive weeks centred on the second week was used for the calculations (the true number of weekly fatalities is shown in Tables 1 and 2).

**Table 2:** Age group and country breakdown of full vaccination uptake, number of deaths observed and averted as well as observed and expected mortality rates per 100,000 population, in the population aged 60 years and older, using the base vaccine effectiveness scenario^a^, by country, 30 WHO European Region countries with sufficient data, weeks 51/2020–45/2021

Country	60–69 year-olds					70–79 year-olds					≥ 80 year-olds				
	Full VU (%)	Deaths observed	Deaths averted	Observed mortality rate per 100,000	Expected mortality rate per 100,000	Full VU (%)	Deaths observed	Deaths averted	Observed mortality rate per 100,000	Expected mortality rate per 100,000	Full VU (%)	Deaths observed	Deaths averted	Observed mortality rate per 100,000	Expected mortality rate per 100,000
Austria	84	676	390	65.4	103.2	80	1,467	675	185.6	271.0	97	3,732	5,581	764.5	1,907.6
Belgium	92	900	1,016	65.7	139.9	94	1,850	2,492	196.9	462.2	90	4,958	4,313	753.4	1,408.7
Croatia	71	225	35	39.8	46.0	76	412	99	113.8	141.2	55	698	117	298.1	348.0
Cyprus	70	108	71	89.7	148.7	81	171	239	217.3	521.1	80	251	344	629.5	1,492.3
Czechia	77	3,434	347	259.2	285.4	86	8,011	1,630	776.9	935.0	84	8,847	3,610	1,987.6	2,798.6
Estonia	69	197	74	116.2	159.9	79	362	235	337.0	555.7	66	731	364	924.4	1,384.6
Finland	90	124	216	17.4	47.7	94	297	671	50.9	166.0	93	586	1,722	188.4	742.1
France	84	6,084	4,786	78.4	140.0	91	11,962	13,533	208.8	445.1	84	29,635	20,396	735.9	1,242.3
Greece	82	2,167	1,998	172.4	331.3	86	3,481	4,250	364.1	808.6	73	6,055	5,927	770.4	1,524.4
Hungary	77	5,110	1,772	391.1	526.8	87	7,276	4,417	859.5	1,381.2	77	8,051	4,077	1,867.4	2,813.0
Iceland	99	1	14	2.7	40.0	100	3	38	12.8	175.1	100	0	0	0.0	0.0
Ireland	100	376	1,189	78.2	325.5	100	837	2,753	253.1	1,085.7	100	1,943	5,132	1,227.9	4,471.3
Israel	89	692	1,532	93.3	299.9	98	1,123	5,271	228.8	1,302.8	97	2,157	8,859	778.6	3,976.4
Italy	86	7,318	2,661	97.8	133.3	88	16,503	5,799	273.7	369.9	92	37,077	27,028	818.7	1,415.6
Latvia	67	572	223	228.7	317.8	67	854	372	524.9	753.5	54	1,376	366	1,282.5	1,623.7
Lithuania	79	709	729	201.0	407.6	82	1,252	1,540	560.7	1,250.4	58	2,194	1,151	1,277.3	1,947.4
Luxembourg	85	52	14	83.7	106.2	87	106	49	279.9	409.3	90	332	202	1,335.1	2,147.3
Malta	96	54	140	93.6	336.3	100	88	167	194.8	564.6	100	163	553	760.2	3,339.4
Montenegro	63	367	142	477.0	661.5	70	537	277	1,328.0	2,013.0	53	495	162	2,311.7	3,068.2
North Macedonia	64	1,368	522	559.2	772.5	72	1,693	933	1,264.5	1,961.4	54	969	319	1,857.9	2,469.6
Norway	95	102	180	17.4	48.1	98	181	707	40.5	198.9	97	399	1,905	174.4	1,007.3
Poland	73	2,049	242	39.6	44.3	81	2,733	747	94.2	120.0	62	3,459	1,003	198.5	256.0
Portugal	98	1,185	1,253	91.6	188.4	99	2,750	3,070	270.1	571.5	98	8,115	9,899	1,189.2	2,639.8
Slovakia	64	2,626	284	377.7	418.6	74	3,693	720	926.2	1,106.8	62	3,500	601	1,948.5	2,283.0
Slovenia	73	345	155	121.1	175.5	86	672	474	378.6	645.7	82	1,781	1,119	1,558.5	2,537.7
Spain	95	4,107	5,722	76.9	184.0	98	8,107	14,387	201.9	560.2	99	21,818	69,406	746.2	3,120.0
Sweden	92	564	291	51.6	78.2	95	1,678	1,142	168.8	283.6	94	4,370	3,337	821.3	1,448.5
Switzerland	80	402	211	41.1	62.7	86	991	495	131.8	197.6	90	3,310	1,937	720.8	1,142.6
United Kingdom (England)	96	9,721	24,719	164.9	584.3	100	18,722	65,338	402.1	1,805.5	93	45,911	67,547	1,641.7	4,057.0
United Kingdom (Scotland)	100	683	5,038	106.6	893.3	100	1,294	8,174	276.2	2,021.2	100	2,608	14,444	977.9	6,393.6
**Total**	**86**	**52,318**	**55,966**	**110.4**	**228.5**	**91**	**99,106**	**140,694**	**285.1**	**689.7**	**88**	**205,521**	**261,421**	**901.3**	**2,047.8**

VU: vaccine uptake; WHO: World Health Organization.

^a^ VE_1_ = 60% and VE_2_ = 95% and time lags of 4 and 3 weeks for first and second vaccination dose, respectively.

The weekly number of deaths averted for each dose was calculated as below for each age group, setting the base scenario for VE against COVID-19 mortality at 60% and 95% for VE_1_ and VE_2_, respectively [11-13]. For single-dose vaccines, the VE of the complete vaccination series was equal to the first dose.


$${Number\;deaths\;averted\;}_{first\;dose,\;w}={Deaths\;}_{observed,\;w}*\frac{{VE}_{1}\left({VU}_{1,\;w-4}-\;{VU}_{2,\;w-3}\right)}{1-\left({VE}_{1}\left({VU}_{1,\;w-4}-{VU}_{2,\;w-3}\right)-{VU}_{2,\;w-3}\right)*{VE}_{2}}\;$$

$${Number\;deaths\;averted\;}_{second\;dose,\;w}=\;{Deaths}_{observed,\;w}*\;\frac{{VU}_{2,\;w-3}*E_{2}}{1-{VE}_{1}\left({VU}_{1,\;w-4}-{VU}_{2,\;w-3}\right)-\;{VU}_{2,\;w-3}*\;{VE}_{2}}\;$$

Here, *w* represents the time delays of 2 and 1 weeks for the development of a full immune response after, respectively, the first and second vaccine dose [14] and the median time from infection to death of 2 weeks [15,16]. The number of deaths averted each week was then added to the number of observed deaths to calculate the total expected number of deaths and cumulative mortality rate per 100,000 population per country.

Using the base VE scenario, we calculated that 51% (n=469,186) of total expected deaths (n=911,302) were averted by vaccination over the study period; ranging from 93% of deaths averted in Iceland to 6% in Ukraine (Table 1). All three countries with 60% or less of their population 60 years and older fully vaccinated by week 45/2021 (Moldova, Romania and Ukraine; Table 1) had a maximum of only 20% expected deaths averted over the study period (Figure 3). On the contrary, all four countries (Israel, Malta, United Kingdom (UK)-England and UK-Scotland) that achieved very high complete vaccination coverage (above 90%) already by week 23/2021 (Figure 1) averted over 65% of total expected deaths by week 45 (Table 1 and Figure 3).

In the 30 countries with more detailed data on age groups allowing for inclusion in age-stratified analyses, the largest number of fatalities were averted after vaccination among people 80 years and older (261,421 fatalities, 57% of total averted deaths). A total of 140,694 (31%) fatalities were averted among 70–79 year-olds and 55,966 (12% of averted deaths) among 60–69 year-olds in the base scenario (Table 2).

## Sensitivity analyses investigating assumed immune response delays and vaccine effectiveness

To investigate the parameters used in the base scenario using VE_1_ (60%) and VE_2_ (95%), we ran sensitivity analyses varying the time to protection and time from infection to death using shorter (3 and 2 weeks, respectively) and longer (5 and 4 weeks, respectively) time ranges.

A sensitivity analysis using median time lags of 4 and 3 weeks was also run using lower bound VE (50% and 70% for first dose and complete series, respectively) and upper bound VE values (70% and 97.5%, respectively). We deemed this range of values representative of the observational studies for the vaccines most frequently used in the countries in this study (Table 1), namely Vaxzevria (AstraZeneca, Oxford, United Kingdom), mRNA-1273 (Moderna, Cambridge (Massachusetts), United States) and Comirnaty (BioNTech-Pfizer, Mainz, Germany/New York, United States) [2].

Using lower and upper bound VE values, we estimated that the number of deaths averted ranged between 129,851 and 733,744, respectively, in people 60 years and older in the 33 countries (Table 1, Supplementary Table S1). The proportion of total estimated deaths averted by vaccination ranged between 23% and 62% according to the VE sensitivity analysis (Supplementary Table S2, where also country-specific estimates are available). There was minimal variation in deaths averted by varying the time lag (Table 1, Supplementary Table S1) . 

## Discussion 

Overall, we estimate the widespread implementation of COVID-19 vaccination programmes for older people has averted a median of 469,186 deaths (sensitivity range: 129,851–733,744) in people 60 years and older in 33 countries (51% of 911,302 expected deaths; sensitivity range: 23–62%). However, this direct impact has been heterogenous (median impact range: 6–93% by country) because of the speed and extent of the vaccination in these eligible groups. Countries with high early uptake (e.g. Israel, Malta, UK-England and UK-Scotland) have substantially reduced predicted mortality, especially in people 80 years and older. Other countries have experienced more limited impact of vaccination to date. Possible explanations are that their programme was implemented more slowly (e.g. Moldova, Romania and Ukraine) or that they achieved higher vaccination levels mainly after a wave of SARS-CoV-2 transmission earlier in 2021 (e.g. Croatia, Czechia and Poland).

A small number of other studies have estimated the number of lives saved from COVID-19 vaccination in older age groups [3,4,17,18], or estimated the difference in life expectancy in three countries [19]. All showed the positive impact of vaccination in saving lives. Our analysis adds to these studies by using a standard approach to compare the estimated direct impact of differential roll-out of COVID-19 vaccine programmes across 33 diverse countries in older adults across the European region.

The analysis contains several limitations and assumptions. Our estimate is conservative as we did not estimate the indirect effect of vaccination or the impact of public health and social measures on mortality by reduction in transmission. The capacity of healthcare systems to respond to the pandemic were not considered here. These primary findings are derived using use a base scenario of vaccine effectiveness and time lag. Using more extreme vaccine effectiveness estimates against mortality in particular scenarios can vary the number of deaths averted as shown in the sensitivity analysis. VE was not differentiated by vaccine manufacturer or type. Based on current, limited data, we assumed that VE against mortality was similar for the SARS-CoV-2 Alpha and Delta variants (Phylogenetic Assignment of Named Global Outbreak (Pango) lineage designation B.1.1.7 and B.1.617.2), the predominant circulating variants of concern during our study period. In addition, we assumed that there has not been any waning in protection against severe disease to date [2]. Differential reporting in mortality surveillance systems was another potential limiting factor, which we assumed to be comparable between countries. Additional third doses of vaccination have not been considered here, as countries had only started to implement them. Many of these limitations and assumptions are likely to have under-estimated the true number of deaths in each country to varying extents. We attempted to account for this by undertaking an 'observed over expected' analysis, estimating the proportion of deaths averted by vaccination to enable a more robust comparison of direct vaccine impact between countries. 

## Conclusion

We show that since the start of COVID-19 vaccination in Europe, the lives of many older adults have been saved through immunisation. Our results highlight large differences between countries in the direct impact of vaccination on COVID-19-related mortality. Early and complete implementation of vaccination of older adults was associated with the largest reduction in expected deaths. It is critically important that all countries rapidly achieve high coverage for these priority groups to prevent further morbidity and mortality, particularly with SARS-CoV-2 transmission rates increasing as Europe moves into the 2021/22 winter period. 

## Supplementary Data

234000 Supplement
