# Author's correction for Euro Surveill. 2021;26(47)

**DOI:** 10.2807/1560-7917.ES.2022.21.220526c

**Published:** 2022-05-26

**Authors:** Eurosurveillance editorial team

**Affiliations:** 1 European Centre for Disease Prevention and Control (ECDC), Stockholm, Sweden

As originally published, equation 2 in the article “Estimated number of deaths directly averted in people 60 years and older as a result of COVID-19 vaccination in the WHO European Region, December 2020 to November 2021” by Meslé et al. was missing one set of brackets. This was corrected on 23 May 2022 at the request of the authors. 

