# Author's correction for Euro Surveill. 2021;26(47)

**DOI:** 10.2807/1560-7917.ES.2022.24.220616c

**Published:** 2022-06-16

**Authors:** Eurosurveillance editorial team

**Affiliations:** 1 European Centre for Disease Prevention and Control (ECDC), Stockholm, Sweden

Equations 1 and 2 in the article “Estimated number of deaths directly averted in people 60 years and older as a result of COVID-19 vaccination in the WHO European Region, December 2020 to November 2021” by Meslé et al. were corrected on 16 June 2022 at the request of the authors. The change refers to the use of brackets in the equations.

