# Supplementary material for: Estimated number of deaths directly averted in people 60 years and older as a result of COVID-19 vaccination in the WHO European Region, December 2020 to November 2021
Source: Euro Surveill. 2021 Nov 25;26(47):2101021. doi: 10.2807/1560-7917.ES.2021.26.47.2101021 (PMC8619871; doi:10.2807/1560-7917.ES.2021.26.47.2101021)
Supplement: Supplement [file 21-01021_PEBODY_Supplement.pdf]

Supplement

Estimated number of deaths directly averted in people 60 years and older as a result of COVID-19 vaccination in the WHO European Region, December 2020 to November 2021 - Appendix

This supplementary material is hosted by *Eurosurveillance* as supporting information alongside the article "Estimated number of deaths directly averted in people 60 years and older as a result of COVID-19 vaccination programmes in the WHO European Region, between December 2020 to and November 2021", on behalf of the authors, who remain responsible for the accuracy and appropriateness of the content. The same standards for ethics, copyright, attributions and permissions as for the article apply. Supplements are not edited by *Eurosurveillance* and the journal is not responsible for the maintenance of any links or email addresses provided therein.

Table S.1: Results of sensitivity analyses according to each alternative Vaccine Effectiveness (VE) and time lag scenarios, showing the total number of deaths averted for each age group (for countries where data is available), expected mortality rate per 100 000 population aged 60 and over, and the % expected deaths averted by vaccination for the population aged 60 years and older, by country, 33 WHO European Region, for weeks 51/2020 to 45/2021.

	**Description**		**Number of deaths averted**				**Expected mortality rate per 100 000 (≥60 year olds)**	**% expected deaths averted by vaccination (≥60 year olds)**
**Scenario**	**VE values**	**Time lags**	**60-69 year olds**	**70-79 year olds**	**≥ 80 year olds**	**≥ 60 year olds**		
**Short time lag**	VE_1= 60%; VE_2= 95%	First dose= 3 weeks; Full coverage= 2 weeks	58,883	147,996	279,467	498,000	776.6	53
**Long time lag**	VE_1= 60%; VE_2= 95%	First dose= 5 weeks; Full coverage= 4 weeks	53,235	134,470	246,086	444,410	732.3	50
**Low VE values**	VE_1= 50%; VE_2= 70%	First dose= 4 weeks; Full coverage= 3 weeks	15,351	32,227	74,710	129,851	472.5	23
**High VE values**	VE_1= 70%; VE_2= 95.7%	First dose= 4 weeks; Full coverage= 3 weeks	83,087	239,430	399,574	733,744	971.4	62

Table S.2: Results of sensitivity analyses according to each alternative Vaccine Effectiveness (VE) scenario, showing the total number of deaths averted for each country, expected mortality rate per 100,000 population aged 60 and over, and the % expected deaths averted by vaccination for the population aged 60 years and older, by country, 33 WHO European Region, for weeks 51/2020 to 45/2021.

	Observed counts and rates		Low VE scenario (VE_1=50% and VE_2=70%)			High VE scenario (VE_1=70% and VE_2=97.5%)		
**Country**	**Deaths**	**Mortality Rate per 100,000 Population**	**Total Averted Deaths**	**Expected Mortality Rate per 100,000 Population**	**% Expected Deaths Averted by Vaccination**	**Total Averted Deaths**	**Expected Mortality Rate per 100,000 Population**	**% Expected Deaths Averted by Vaccination**
Austria	5,875	254.1	1,801	332.0	23	9,647	671.4	62
Belgium	7,708	259.8	2,633	348.5	25	9,887	592.9	56
Croatia	1,335	114.9	161	128.8	11	283	139.3	18
Cyprus	530	221.8	304	349.0	36	736	529.8	58
Czechia	20,292	724.5	2,802	824.5	12	6,419	953.7	24
Estonia	1,290	362.4	361	463.8	22	743	571.1	37
Finland	1,007	62.7	749	109.3	43	3,555	284.0	78
France	47,681	272.2	16,751	367.8	26	47,291	542.1	50
Greece	11,703	390.2	5,691	579.9	33	13,644	845.1	54
Hungary	20,437	790.9	5,273	994.9	21	11,834	1,248.8	37
Iceland	4	5.4	7	14.9	64	102	143.8	96
Ireland	3,156	325.5	1,219	451.2	28	18,397	2,222.7	85
Israel	3,972	263.1	3,854	518.5	49	23,253	1,803.6	85
Italy	60,898	337.5	12,971	409.4	18	44,709	585.3	42
Latvia	2,802	538.7	576	649.4	17	1,025	735.8	27
Lithuania	4,155	555.6	1,652	776.5	28	3,800	1,063.7	48
Luxembourg	490	392.4	107	478.1	18	318	647.1	39
Malta	305	245.4	147	363.7	33	1,619	1,548.0	84
Moldova	3,584	470.3	349	516.1	9	550	542.5	13
Montenegro	1,399	1,008.0	330	1,245.7	19	622	1,456.1	31
North Macedonia	4,030	935.7	976	1,162.3	19	1,939	1,385.9	32

Norway	682	54.1	569	99.2	45	4,306	395.5	86
Poland	8,241	83.9	1,206	96.2	13	2,194	106.3	21
Portugal	12,050	402.4	2,481	485.2	17	24,980	1,236.6	67
Romania	30,250	606.3	5,006	706.6	14	7,861	763.8	21
Slovakia	9,819	771.0	955	846.0	9	1,763	909.4	15
Slovenia	2,798	485.2	781	620.6	22	1,991	830.4	42
Spain	34,032	277.1	14,602	396.0	30	163,729	1,610.5	83
Sweden	6,612	252.3	1,457	308.0	18	6,419	497.3	49
Switzerland	4,703	214.9	1,025	261.7	18	3,122	357.5	40
Ukraine	51,337	496.5	2,208	517.8	4	3,242	527.8	6
United Kingdom (England)	74,354	557.1	37,170	835.6	33	257,344	2,485.3	78
United Kingdom (Scotland)	4,585	333.3	3,677	600.6	45	56,420	4,434.8	92
**Total**	**442,11**	**365.2**	**129,851**	**472.5**	**23**	**733,744**	**971.4**	**62**
